# ﻿Looking back at the 12^th^ International Symposium on Terrestrial Isopod Biology

**DOI:** 10.3897/zookeys.1225.147808

**Published:** 2025-02-05

**Authors:** Ivan Hadrián Tuf, Karel Tajovský, Stefano Taiti

**Affiliations:** 1 Department of Ecology and Environmental Sciences, Faculty of Science, Palacký University Olomouc, Šlechtitelů 27, 77900 Olomouc, Czech Republic Palacký University Olomouc Olomouc Czech Republic; 2 Biology Centre CAS, Institute of Soil Biology and Biogeochemistry, Na Sádkách 7, 37005 České Budějovice, Czech Republic Biology Centre CAS, Institute of Soil Biology and Biogeochemistry České Budějovice Czech Republic; 3 Research Institute on Terrestrial Ecosystems, National Research Council (IRET-CNR), Via Madonna del Piano 10, 50019 Sesto Fiorentino (Florence), Italy Research Institute on Terrestrial Ecosystems, National Research Council Florence Italy

It was Professor Warburg who originally suggested the idea of organizing a meeting of those specialists who, in various fields, were engaged in the study of terrestrial isopods (Oniscidea). Thanks to the efforts of Stephen L. Sutton and David M. Holdich, the first symposium devoted to this group of invertebrates was held in 1983 under the auspices of the Zoological Society of London. This symposium was followed by the publication of the first comprehensive collection of papers bringing together new knowledge about the biology, ecology, and behaviour of terrestrial isopods in one place. This initial impulse was subsequently followed by further meetings at irregular intervals, which gave rise to what is now a relatively long tradition of symposia dedicated to terrestrial isopods (Fig. 1). Proceedings based on the symposia were usually published in the year following the meetings, traditionally containing selected published papers. Their number varied as the rules for accepting manuscripts and the practice of publishing results in the wider scientific community changed (Fig. 2). At a certain point in time, the era of monographic proceedings came to an end, and scientists were increasingly expected to publish mainly in prestigious scientific journals. However, this was accompanied by changes in the funding of proceedings, with costs shifting from all participants to individual authors. Starting with the 8^th^ symposium, it became a tradition that the proceedings were published as a special issue of the peer-reviewed open-access journal ZooKeys. We thank the editorial board of the journal for this long-standing favour and support.

**Figure 1.**
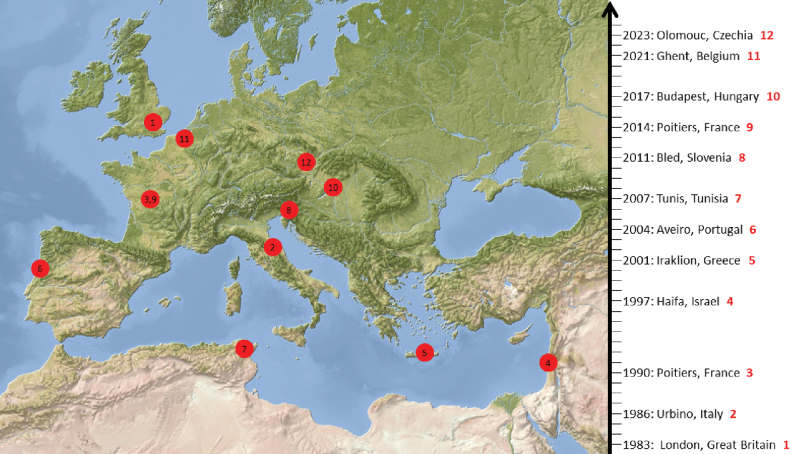
Spatio-temporal distribution of all symposia on terrestrial isopods. The red number following the year and place corresponds to the spots in the map.

**Figure 2.**
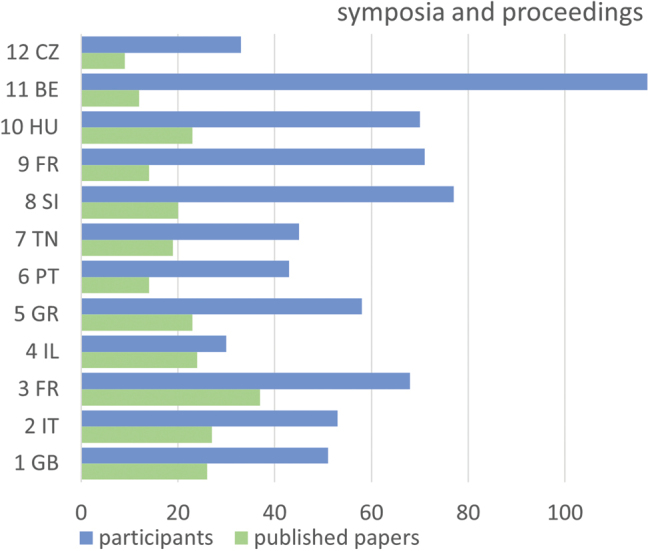
Symposia on terrestrial isopods in numbers. Numbers of participants and published papers in individual proceedings.

A change in the behaviour of scientists was linked to the COVID-19 pandemic, which led to the 11^th^ Symposium being held in Ghent only online. This option, excellently organised by the colleague of the Spinicornis (the Belgian Terrestrial Isopod Group), was used by 117 participants, an absolute record, but only 12 contributions (again a record at the time) were published in the subsequent proceeding volume.

The last ISTIB, the 12^th^ in order, was held in Olomouc, the Czech Republic under the joint auspices of the Biology Centre CAS and Faculty of Science, Palacký University Olomouc (Fig. 3). A total of 30 participants from 11 countries attended the meeting (Fig. 4), which was conducted in a pleasantly intimate atmosphere. Altogether, 21 lectures were presented and 14 posters were displayed. The result of the symposium is published in a proceedings volume that contains nine papers on various aspects of the biology, ecology, taxonomy, and behaviour of terrestrial isopods.

**Figure 3.**
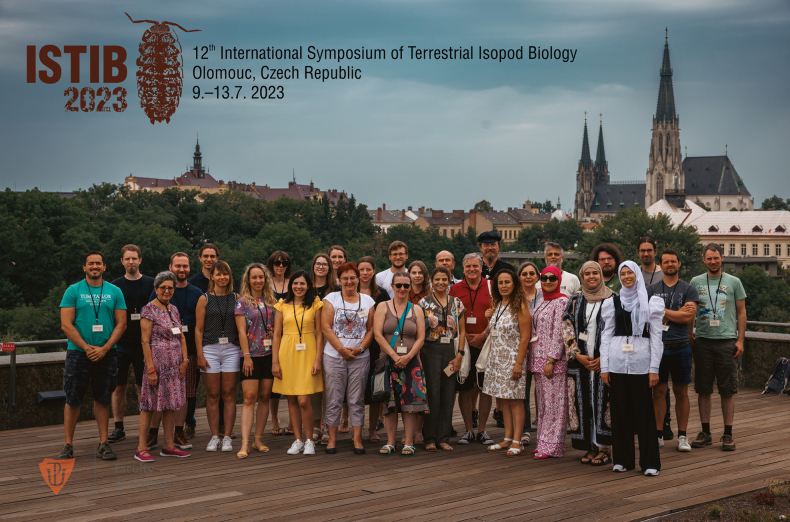
Congress photo of the 12^th^ International Symposium on Terrestrial Isopod Biology. The logo of this meeting, made by Eva Tajovská, depicted the species *Protracheoniscuspolitus* (C. Koch, 1841), one of the 18 taxa of terrestrial isopods described from the Czech Republic (photograph Ota Blahoušek).

**Figure 4.**
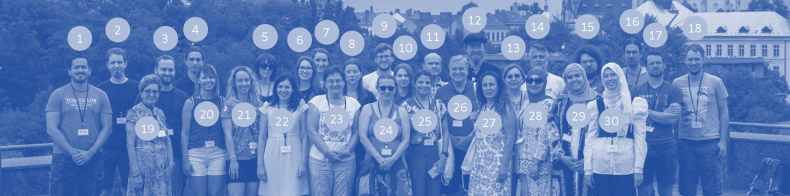
Participants of the 12^th^ ISTIB. 1 – Ivanklin Soares Campos-Filho; 2 – Urban Bogataj; 3 – Miloš Vittori; 4 – Pietro Gardini; 5 – Jessica Thomas Thorpe; 6 – Tina Petrišič; 7 – Ana Nagode; 8 – Petra Štern; 9 – Martin Martinka; 10 – Barbora Ďurajková; 11 – Karel Tajovský; 12 – Ivan Hadrián Tuf; 13 – Katalin Szlavecz; 14 – Spyros Sfenthourakis; 15 – Gert Arijs; 16 – Pepijn Boeraeve; 17 – Pallieter De Smedt; 18 – Stijn Segers; 19 – Faouzia Charfi-Cheikhrouha; 20 – Polona Mrak; 21 – Katja Kunčič; 22 – Mariana Nunes; 23 – Elisabeth Hornung; 24 – Jana Bedek; 25 – Karima Nasri Ammar; 26 – Stefano Taiti; 27 – Lamia Medini-Bouaziz; 28 – Nermine Laifi; 29 – Imane Benchana; 30 – Salsabil Abidi.

Research on the biology of terrestrial isopods is still ongoing as there are many gaps to fill in their taxonomy, phylogeny, physiology, ecology, and behaviour. Currently, more than 4,100 species are known worldwide, with an increase of approximately 500 species during the last 20 years. However, many regions, particularly in the tropics, remain poorly studied, and many new taxa in various museum collections are still awaiting description. We look forward to the next symposium to learn more about this fascinating and important group of soil arthropods, our favourite woodlice, also called sowbugs, pill bugs, slaters, roly polies, butchy boys, or potato bugs.

## ﻿Proceedings published from the ISTIB symposia:

Sutton SL, Holdich DM (Eds) (1984) The Biology of Terrestrial Isopods. The Proceedings of a Symposium held at the Zoological Society of London on 7^th^ and 8^th^ of July 1983. (Symposia of the Zoological Society of London 53). Clarendon Press, Oxford, 518 pp.

Ferrara F, Argano R, Manicastri C, Schmalfuss H, Taiti S (Eds) (1989) Proceedings of the Second Symposium on the Biology of Terrestrial Isopods (Urbino, Italy, 10–12 September 1986). Monitore Zoologico Italiano, NS Monogr. 4, 512 pp.

Juchault P, Mocquard JP (Eds) (1991) Third Symposium on the Biology of Terrestrial Isopods, Poitiers, France, 10–12 July 1990. Université de Poitiers, France, 222 pp.

Hassall M, Hornung E, Warburg MR (Eds) (1998) Oniscidean Isopods. Proceedings of the 4^th^ Symposium on the Biology of Terrestrial Isopods, Haifa. Israel Journal of Zoology 44: 1–250.

Sfenthourakis S, de Araujo PB, Hornung E, Schmalfuss H, Taiti S, Szlávecz K (Eds) (2003) The biology of terrestrial isopods, V. Oniscidea rolling into the new millennium: Proceedings of the 5^th^ International Symposium on the Biology of Terrestrial Isopods, Irakleio (Iraklion), Crete, Greece, 19–23 May 2001. (Crustaceana Monographs 2). Brill Academic Publisher, Leiden, 386 pp.

Loureiro S, Soares AMVM, Araujo PB, Sfenthourakis S, Hornung E, Zimmer M, Schmalfuss H, Taiti S (Eds) (2005) The biology of terrestrial isopods, VI. Proceedings of the 6^th^ International Symposium on the Biology of Terrestrial Isopods. European Journal of Soil Biology 41(3–4): 55–167.

Zimmer M, Charfi-Cheikhrouha F, Taiti S (Eds) (2008) Proceedings of the international symposium on terrestrial isopod biology: ISTIB-07. Shaker-Verlag, Aachen, 176 pp, 5 appendices.

Štrus J, Taiti S, Sfenthourakis S (Eds) (2012) Advances in Terrestrial Isopod Biology. ZooKeys 176: 1–296.

Taiti S, Hornung E, Štrus J, Bouchon D (Eds) (2015) Trends in Terrestrial Isopod Biology. ZooKeys 515: 1–206.

Hornung E, Taiti S, Szlavecz K (Eds) (2018) Isopods in a Changing World. ZooKeys 801: 1–518.

De Smedt P, Taiti S, Sfenthourakis S, Campos-Filho IS (Eds) (2022) Facets of terrestrial isopod biology. ZooKeys 1101: 1–212.

Tuf IH, Tajovský K, Taiti S (Eds) (2025) The Biology of Terrestrial Isopods, XII. ZooKeys 1225: 1–154.

## ﻿Additional information

### Author ORCIDs

Ivan Hadrián Tuf https://orcid.org/0000-0003-0250-0482

Karel Tajovský https://orcid.org/0000-0002-3619-3815

Stefano Taiti https://orcid.org/0000-0002-4909-6037

### Data availability

All of the data that support the findings of this study are available in the main text.

